# One Health Surveillance Highlights Circulation of Viruses with Zoonotic Potential in Bats, Pigs, and Humans in Viet Nam

**DOI:** 10.3390/v15030790

**Published:** 2023-03-20

**Authors:** Alice Latinne, Nguyen Thi Thanh Nga, Nguyen Van Long, Pham Thi Bich Ngoc, Hoang Bich Thuy, PREDICT Consortium, Nguyen Van Long, Pham Thanh Long, Nguyen Thanh Phuong, Le Tin Vinh Quang, Nguyen Tung, Vu Sinh Nam, Vu Trong Duoc, Nguyen Duc Thinh, Randal Schoepp, Keersten Ricks, Ken Inui, Pawin Padungtod, Christine K. Johnson, Jonna A. K. Mazet, Chris Walzer, Sarah H. Olson, Amanda E. Fine

**Affiliations:** 1 Wildlife Conservation Society, Viet Nam Country Program, Hanoi 11111, Viet Nam; 2 Wildlife Conservation Society, Health Program, Bronx, NY 10460, USA; 3 Department of Animal Health, Ministry of Agricultural and Rural Development of Viet Nam, Hanoi 11519, Viet Nam; 4 Regional Animal Health Office No. 6, Ho Chi Minh City 72106, Viet Nam; 5 National Institute of Hygiene and Epidemiology, Ministry of Health, Hanoi 11611, Viet Nam; 6 Diagnostic Systems Division, U.S. Army Medical Research Institute of Infectious Diseases, Frederick, MD 21702, USA; 7 Food and Agriculture Organization of the United Nations (FAO), Country Office for Viet Nam, Hanoi 11112, Viet Nam; 8 One Health Institute, School of Veterinary Medicine, University of California, Davis, CA 95616, USA; 9 Research Institute of Wildlife Ecology, University of Veterinary Medicine Vienna, 1210 Vienna, Austria

**Keywords:** One Health, bats, pigs, zoonoses, livestock, coronavirus, paramyxovirus, influenza, spillover, surveillance

## Abstract

A One Health cross-sectoral surveillance approach was implemented to screen biological samples from bats, pigs, and humans at high-risk interfaces for zoonotic viral spillover for five viral families with zoonotic potential in Viet Nam. Over 1600 animal and human samples from bat guano harvesting sites, natural bat roosts, and pig farming operations were tested for coronaviruses (CoVs), paramyxoviruses, influenza viruses, filoviruses and flaviviruses using consensus PCR assays. Human samples were also tested using immunoassays to detect antibodies against eight virus groups. Significant viral diversity, including CoVs closely related to ancestors of pig pathogens, was detected in bats roosting at the human–animal interfaces, illustrating the high risk for CoV spillover from bats to pigs in Viet Nam, where pig density is very high. Season and reproductive period were significantly associated with the detection of bat CoVs, with site-specific effects. Phylogeographic analysis indicated localized viral transmission among pig farms. Our limited human sampling did not detect any known zoonotic bat viruses in human communities living close to the bat cave and harvesting bat guano, but our serological assays showed possible previous exposure to Marburg virus-like (Filoviridae), Crimean–Congo hemorrhagic fever virus-like (Bunyaviridae) viruses and flaviviruses. Targeted and coordinated One Health surveillance helped uncover this viral pathogen emergence hotspot.

## 1. Introduction

One Health surveillance is described as “the systematic collection, validation, analysis, interpretation of data and dissemination of information collected on humans, animals and the environment to inform decisions for more effective, evidence- and system-based health interventions” [1] and has been identified as a cost-effective approach to the early detection and timely control of zoonotic pathogens that could cause pandemics [2]. Since the COVID-19 crisis, calls for integrated One Health surveillance as a key to pandemic prevention and preparedness have been highlighted and reemphasized by many scientists and expert panels [3,4,5,6,7]. Although the need to align surveillance for viruses with pandemic potential across possible animal reservoir hosts (wild and domestic species) and at-risk populations of humans is clear, implementing cross-sectoral One Health surveillance at sites of interspecies and high-risk interfaces for viral spillovers is often complex. The restricted mandates, resources, and surveillance priorities of most national animal and public health institutions, coupled with limited published examples of attempted implementation of One Health surveillance strategies to learn from, exacerbate the challenges. In this cross-sector One Health study in Viet Nam, we broadly employed the two surveillance strategies that have been identified as necessary to effectively detect or preempt spillover: (1) targeting locations where spillovers are most likely to occur, and (2) coordinating concurrent surveillance in wildlife, domestic animals and people.

Our study involved screening biological samples from bats, pigs, and humans for five viral families with known spillover potential including coronaviruses, paramyxoviruses, and influenza viruses as well as filoviruses and flaviviruses. Surveillance was coordinated around bat guano harvesting sites and pig farming operations which are considered high-risk interfaces for viral spillover between animals and people. Viet Nam has been the world’s sixth-largest pork producer during the last decade [8], and high densities of intensively and extensively raised pigs occur across the country with pork representing the main part of total meat production [9]. Although the size of pig farms vary, small scale farm households with 1–4 pigs represent the majority of the pig production system in the country [9]. To operationalize this One Health surveillance approach at the national level, the United States Agency for International Development’s PREDICT project activities, which sought to strengthen capacity for detection of viruses of pandemic potential in Viet Nam, were aligned with the existing national influenza surveillance program in poultry and pigs, as well as the severe acute respiratory illness (SARI) surveillance programs in humans in Viet Nam, under an initiative known as the Longitudinal Influenza Surveillance Network (LISN). The LISN initiative’s communication and consultation process was co-led by the Department of Animal Health (DAH), Ministry of Agriculture and Rural Development (MARD), and the General Department of Preventive Medicine, Ministry of Health with technical support from PREDICT, the United Nations Food and Agriculture Organization (FAO), the World Health Organization (WHO), and the US Centers for Diseases Control and Prevention.

Of particular concern in the infectious disease emergence context of Asia and Viet Nam are coronaviruses (CoVs) which are characterized by strong host plasticity, as their genomic features predispose them to recombination and cross-species transmission [10]. The coronavirus family contains numerous emerging and zoonotic viral pathogens of high importance for human and livestock health. Bats harbor a large diversity of CoVs belonging to the α-CoV and β-CoV genera [11,12] and have been identified as the evolutionary precursors of most CoVs infecting humans [13], including Severe Acute Respiratory Syndrome (SARS)-CoV-1 and SARS-CoV-2 [14,15], Middle East Respiratory Syndrome (MERS)-CoV [16], HCoV-NL63 [17], and HCoV-229E [18]. Paramyxoviruses (PmVs) also include several significant zoonotic pathogens in Southeast Asia, with a majority of them originating from bats [19,20].

Besides humans, emerging CoVs and PmVs also cause various diseases in domestic pigs. Several CoV and PmV spillovers from bats to pigs occurred in Asia and in Australia in the last two decades and had considerable economic impact in the region. In 1997, Menangle virus (MenPV) emerged in swine farms in Australia, causing reproductive disorders in pigs. MenPV was subsequently isolated from urine samples of the black flying fox *Pteropus alecto*, suggesting a bat origin for this virus [21]. A year later, in 1998, Nipah virus (NiV) spilled over from fruit bats (flying foxes) to pigs in Peninsular Malaysia, causing respiratory illness in pigs [22,23]. Over 800,000 pigs were culled from the outbreak area in 1999 before the epidemic ended [24]. During the emergence of both NiV and MenPV, infected pigs subsequently contaminated workers in swine farms, leading to at least 105 human deaths in the case of NiV [22,25]. More recently, swine acute diarrhea syndrome coronavirus (SADS-CoV) emerged in Guangdong province, China, in 2016, where it caused fatal swine disease outbreaks and high piglet mortality in numerous pig farms in southern China [26,27]. Bat surveillance data from China showed that SADS-CoV was very closely related to HKU2-CoV, a CoV infecting mostly horseshoe bats in the same geographic region [26]. Pigs are also intermediate hosts for influenza viruses and play an important role in the evolution and diversification of influenza A viruses of human and avian origin in Asia [28]. Repeated spillovers of influenza viruses from human to swine were detected in Viet Nam, highlighting the risk for influenza reassortments in pigs [29]. These examples not only highlight the significance of the bat–pig–human interfaces in Asia in the emergence of CoVs, PmVs and influenza viruses, but also illustrate why a One Health surveillance approach is critical to understanding viral disease emergence at these interfaces and the importance of informing effective systems-based health interventions. Flaviviruses and filoviruses were also integrated into our surveillance strategy, as these viral families host high-impact zoonotic viruses, such Zika and Ebola viruses, and their prevalence and ecology in Asia remain poorly understood.

Bat guano harvesting is a common practice in Viet Nam and Southeast Asia, where bat guano is used as natural plant fertilizer [30]. Bat guano is traditionally harvested in caves with large roosting colonies of cave-dwelling insectivorous bats [31]. Inside caves, the bat guano harvesters and cave owners are exposed to overhead droppings and regularly walk through piles of bat feces with uncovered feet. As much as 70 tons of guano can be harvested annually in a single cave [31]. In Viet Nam and Cambodia, bat guano is also collected under artificial bat roosts constructed with a concrete base and pillars topped with fronds of coconut palm which attract foliage-dwelling bats [32]. These artificial roosts are called bat guano farms, and they are often located near human dwellings, where domestic animals and crops are raised. In this study, we investigated zoonotic disease spillover risk related to guano harvesting at Tan Lap cave, a natural bat roosting site in Huu Lung district within Lang Son province, where guano is collected by local harvesters from June to September and from December to March, and at constructed bat guano farms where the bat guano is collected and harvested by hand in Dong Thap and Soc Trang provinces. We also conducted a serological survey in a small group of human individuals in frequent contact with bat guano (bat guano harvesters) at Tan Lap cave.

In this study, we implemented a One Health surveillance approach to screen biological samples from bats, pigs and humans for five viral families with epidemic or pandemic potential. Consistent methods for identifying viruses were applied to specimens from bat, pig, and human populations at these high-risk interfaces for zoonotic disease transmission, and data were integrated in final analyses. The aim of the study was to identify potential spillover events at high-risk interfaces for zoonotic disease transmission and improve our understanding of the epidemiology and evolution of CoV and PmV viruses identified in Viet Nam using phylogeographic tools.

## 2. Material and Methods

### 2.1. Sample Collection

**Figure 1:**
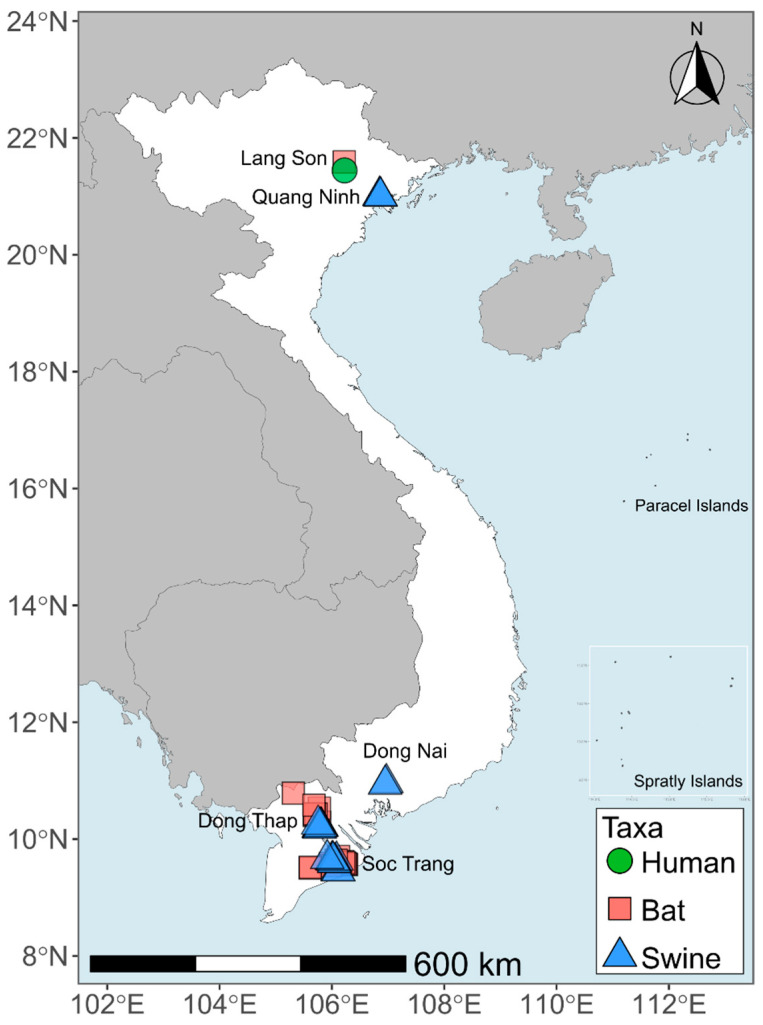
Map of sampling locations in targeted provinces in Viet Nam.

The provinces of Quang Ninh, Dong Thap, Dong Nai, Soc Trang, and Lang Son were selected for piloting the coordinated One Health surveillance in Viet Nam by the cross-sectoral stakeholders involved in the LISN project (Figure 1). Sites were selected to capitalize on existing surveillance efforts in human, wildlife, and livestock populations, and to have representative sites in both northern and southern Viet Nam.

Bat samples from 21 bat–human contact sites were collected in 2013–2014 (as described in [32]) and/or 2017–2018 as part of the PREDICT project [33]. The sites included artificially constructed bat roosts to facilitate guano collection located in Dong Thap and Soc Trang provinces, the Tan Lap natural bat cave located in Lang Son province, and one natural fruit bat roost that is protected from human threats due to its location within the grounds of a Buddhist pagoda located in Soc Trang province. At each site, bat fecal and/or urine samples were collected on clean plastic sheets placed under bat roosts one or two hours before sample collection [32]. We also collected a small number of oral and rectal swabs from live-captured bats at the natural fruit bat roost and fresh carcasses of bats found dead at a bat guano farm. In total, 1035 fecal samples, 55 urine samples, 19 oral swabs and 22 rectal swabs from bats were collected in these three provinces (Table S1).

In 2015–2016, nasal swabs were collected from 123 pigs in 12 farms located in Dong Thap province and from 62 pigs in 10 farms from Soc Trang province (Table S1) (Figure 1) [33]. In 2017, we collected oral swabs from 60 pigs in two farms in Dong Nai province, 60 pigs in two farms in Dong Thap province and 60 pigs in four farms in Quang Ninh province (Table S1) [33]. In 2018, an additional set of oral swabs was collected from 60 pigs in Dong Nai province and 60 pigs in Dong Thap province under the LISN initiative (Table S2). Herd size of most farms where samples were collected was between 100 and 320 pigs, but one farm in Dong Nai and one farm in Quang Ninh province had 1200 and 700 pigs, respectively.

Human samples were also collected from 30 individuals, either guano harvesters working in or community members living in close proximity to the Tan Lap cave in July 2017 (Table S1) (Figure 1) [33]. After consent was given, an oral swab and whole blood (1.5–2.0 mL) were collected from all participants. The whole blood sampled from each participant was then centrifuged at 1800× *g* and the serum (0.5–1.0 mL) collected.

For all samples other than the human serum, duplicates were collected and stored in cryotubes, one containing 1 mL of RNAlater (RNA stabilization reagent, Qiagen) or Trizol and one containing 1 mL VTM (Viral Transport Medium) and stored in liquid nitrogen in the field before being transported to the laboratory for storage at −80 °C. Bat and pig samples were tested by the Regional Animal Health Offices No. 6 (RAHO6/Ho Chi Minh) and No. 2 (RAHO2/Hai Phong) laboratories, while human and bat samples collected in Lang Son province were tested at the National Institute of Hygiene & Epidemiology (NIHE) in Ha Noi.

The study and sampling activities for specified dates and locations were approved by the Department of Animal Health of the Ministry of Agriculture and Rural Development, and animal sampling protocols were approved by the Institutional Animal Care and Use Committee at the University of California at Davis (protocol number 16048). The Institutional Review Board of the University of California at Davis (#804522) and at NIHE, Ministry of Health (Ref: VN01057-20/2016) in Viet Nam approved the human study protocol.

### 2.2. Bat Species Identification

Fruit bats from the natural bat roost in Soc Trang province were identified using morphological criteria and belonged to the species *Pteropus lylei* and *Cynopterus* sp. A subset of bat samples collected at the bat cave in Lang Son province and guano farms in Dong Thap province was barcoded by sequencing a short fragment of the mitochondrial gene *cytochrome b* (cytb). PCRs were performed using DNA extracted from fecal samples with cytb primers and protocols developed by Townzen et al. [34]. We were able to successfully barcode 38 of these bat samples and two bat species were identified, each restricted to a single province: the wrinkle-lipped free-tailed bat, *Chaerephon plicatus*, in the cave in Lang Son and the lesser Asiatic yellow bat, *Scotophilus kuhlii*, in the bat guano farms in Dong Thap. Other bat species may have been present at those sites but were not identified in our subset of barcoded samples.

### 2.3. Viral Screening

A total of 485 pig samples (nasal and oral swabs), 891 bat samples (fecal and urine samples, oral and rectal swabs) and 60 human samples (whole blood and oral swabs) (Table 1) were tested for: coronaviruses (CoVs), paramyxoviruses (PmVs), influenza A viruses (IAV), flaviviruses (FlaviVs) and filoviruses (FiloVs). An additional sample set including 240 bat samples from guano farms in Dong Thap province collected in December 2017 and June 2018 were tested for CoVs only as resources were not available to test them for all five viral families (Table 1 and Table S1). 

RNA was extracted from all samples (RNA MiniPrep Kit, Sigma-Aldrich) and cDNA transcribed (SuperScript III First Strand cDNA Synthesis System, Invitrogen). CoV RNA was detected using two broadly reactive consensus nested-PCR assays targeting the RNA dependent RNA polymerase (RdRp) gene [35,36]. A consensus hemi-nested PCR assay targeting the polymerase (Pol) gene (primers PAR-F1, PAR-F2, PAR-R) was used to detect paramyxovirus RNA [37]. Two assays targeting the M gene [38] and RNA-directed RNA polymerase subunit PB1 gene (Liang E, unpublished assay) were used to detect classic and newly identified divergent influenza A virus RNA. Samples were also screened for filoviruses and flaviviruses using PCR assays targeting the L gene [39] and NS5 gene [40], respectively. All PCR protocols and cycle conditions were performed as described in [41]. Synthetic plasmids containing the primer binding sites for all assays were used as positive controls. Distilled water was used as a negative control and included in each test batch. PCR products were visualized using 1.5% agarose gels, and bands of the correct size were excised, TA-cloned and sequenced by Sanger dideoxy sequencing using the same primers as for amplification by Macrogen Inc. (Seoul, Republic of Korea). Data on CoV screening of bat samples collected in 2013–2014 were previously reported in Huong et al. [32].

In addition to these broadly reactive consensus PCR tests, 539 bat samples collected in the guano farms and the bat cave were additionally screened specifically for Sarbecoviruses and SARS-CoV-2 using a RT-PCR assay targeting the E gene and RdRp gene [42] and cDNA previously obtained for the consensus PCR assays (Table S1). Samples with positive results for either the E gene or RdRp gene assay were then screened using conventional PCR and primers specifically designed to detect pangolin Sarbecoviruses [43].

**Table 1:** Summary of positive samples by taxa, sample type and viral family collected under the PREDICT (Table S1) and LISN (Table S2) initiatives.

Taxa	Sample Type	CoVs	PmVs	Influenza	Flavi	Filo	Viral Species
Bats	Guano and feces	32.7% (339/1035) ^a^	1.5% (13/835)	0% (0/833)	0% (0/835)	0% (0/833)	- PREDICT_CoV-17 (n = 4) - PREDICT_CoV-35 (n = 45) - Bat coronavirus 512/2005 (n = 294) - PREDICT_CoV-47 (n = 12) - PREDICT_CoV-82 (n = 2) - PREDICT_CoV-99 (n = 1) - Porcine epidemic diarrhea virus (n = 1) ^b^ - Alphacoronavirus 1 (n = 1) - PREDICT_PmV-13 (n = 5) - PREDICT_PmV-63 (n = 3) - PREDICT_PmV-66 (n = 5)
	Urine	0% (0/55)	16% (4/25)	0% (0/25)	0% (0/25)	0% (0/25)	- PREDICT_PmV-13 (n = 1) - PREDICT_PmV-63 (n = 1) - PREDICT_PmV-67 (n = 2)
	Oral swabs	10.5% (2/19)	0% (0/14)	0% (0/14)	0% (0/14)	0% (0/14)	- Bat coronavirus 512/2005 (n = 2)
	Rectal swabs	18.2% (4/22)	0% (0/17)	0% (0/17)	0% (0/17)	0% (0/17)	- Bat coronavirus 512/2005 (n = 4)
	Total	30.5% (345/1131)	1.9% (17/888)	0% (0/888)	0% (0/888)	0% (0/888)	
Pigs	Nasal swabs	0% (0/185)	1.6% (3/185)	8.1% (15/185)	0% (0/185)	0% (0/185)	- Porcine Parainfluenzavirus 1 (n = 3) - Influenza A (n = 15)
	Oral swabs	40% (120/300)	2.3% (7/300)	3% (9/300)	0% (0/300)	0% (0/300)	- Alphacoronavirus 1 (n = 28) - Betacoronavirus 1 (n = 97) - Porcine Parainfluenzavirus 1 (n = 7) - Influenza A (n = 9)
	Total	24.7% (120/485)	2.1% (10/485)	4.9% (24/485)	0% (0/485)	0% (0/485)	
Humans	Whole blood	0% (0/30)	0% (0/30)	0% (0/30)	0% (0/30)	0% (0/30)	
	Oral swabs	0% (0/30)	0% (0/30)	0% (0/30)	0% (0/30)	0% (0/30)	
	Total	0% (0/60)	0% (0/60)	0% (0/60)	0% (0/60)	0% (0/60)	

^a^ Includes 338 positive samples detected by the broadly reactive CoV consensus PCR [35,36] and one sample detected using the Sarbecovirus/SARS-CoV-2 assays [42,43]. ^b^ Porcine epidemic diarrhea virus was detected using the Sarbecovirus/SARS-CoV-2 assays [42,43] and was not detected by the broadly reactive CoV consensus PCR [35,36].

### 2.4. Phylogenetic Analysis

For sequence analysis and virus classification, operating taxonomic units were defined with a cut off of 90% sequence identity, i.e., groups sharing ≥ 90% identity to a sequence already in GenBank were given the same name as the matching sequence and virus sequences that shared less than 90% identity to a known sequence were labeled sequentially as PREDICT_CoV/PmV-1, -2, -3, etc. [44]. Subsets of sequences representing either the whole CoV or PmV diversity detected in our samples were selected for our phylogenetic analysis. Representatives of all CoV and PmV species infecting mammals or birds and currently recognized by the ICTV were also included in the dataset (Tables S3 and S4). Sequences of porcine deltacoronavirus (GenBank accession numbers KP757891 and MK993519) and Sunshine virus (GenBank accession number JN192445) were used as outgroups in our CoV and PmV phylogenetic trees, respectively.

Phylogenetic reconstructions were performed on the concatenated dataset, including two fragments of the RdRp gene [35,36], for CoVs and on the pol gene dataset for PmVs using the Maximum Likelihood (ML) approach. ML analyses were performed using PhyML 3.0 [45]. The transition/transversion ratio, the proportion of invariable sites and the gamma distribution parameter were estimated and the substitution model was automatically selected [46]. The starting tree was determined by BioNJ analysis of the datasets. Robustness of the tree was assessed by 1000 bootstrap replicates.

### 2.5. Phylogeographic Analysis

The phylogeographic structures of viruses most commonly detected in our bat and pig samples, including bat CoV 512/2005 (RdRp gene, [36]), PREDICT_CoV-35 (RdRp gene, [36]), Betacoronavirus 1 (BetaCoV1; RdRp gene, [36]), porcine parainfluenza virus 1 (PPV1; pol gene) and influenza A virus (IAV; PB1 gene), were investigated with median-joining networks reconstructed using PopART 1.7 [47]. Sequences from the same viral species from other regions of the world were included in our datasets for analysis (Tables S3–S5).

We also used a Bayesian discrete phylogeographic model available in BEAST 1.10.2 [48] and ancestral host reconstruction to infer the spatiotemporal dynamics of bat CoVs related to the bat coronavirus 512/2005 in Southeast Asia. Sampling years and months were used as tip dates and sampling provinces were used as character states. Preliminary analyses were run to select the best combination of priors. The best-fitting models, including an HKY substitution model with two codons partitions ((1 + 2), 3) and a strict molecular clock and a constant population size coalescent model, were used to perform the final analysis, which was run for 1 × 10^8^ generations with sampling every 1 × 10^4^ steps. An asymmetric trait substitution model was applied. All BEAST computations were performed on the CIPRES Science Gateway Portal [49]. Convergence of the chain was assessed in Tracer 1.7 [50] so that the effective sample size (ESS) of all parameters was >200 after removing at least 10% of the chain as burn-in. MCC trees annotated with discrete traits were generated in TreeAnnotator and visualized using the software SpreaD3 [51].

### 2.6. Statistical Analysis of Seasonal Effect on CoV Shedding

The effects of the season (dry and wet, with dry season as reference category) and reproductive cycle (birth season and no birth season, with birth season as reference category) on the CoV positivity rate in guano farms (Dong Thap and Soc Trang provinces) and in the bat cave (Lang Son province) were assessed with univariate generalized linear models (GLM) using the R package lme4 [52] in R 4.0.2 [53]. The same code as in [32], available at https://doi.org/10.1371/journal.pone.0237129.s005 (accessed on 17 March 2023), was used to perform the analysis. 

In the southern provinces of Dong Thap and Soc Trang, the wet season occurs from May 1st to November 30th, while the dry season occurs from December 1st to April 30th [32]. The constructed bat guano farms located in these two provinces were visited five times for sample collection, three times during the wet season (October 2013, July 2017, June 2018) and two times during the dry season (January 2013, December 2017; Table 2). In this region, the birth and lactation season of the Lesser Asiatic Yellow bat (*Scotophilus kuhlii*) is reported to be April-June [54,55]; therefore, the samples collected in July 2017 and June 2018 were included in the “birth + lactation season” while all other samples were considered “no birth season” (Table 2).

In the northern province of Lang Son, the wet season is shorter than in the South and occurs from 1st May to 30th September, while the dry season lasts from 1st October to 30th April [32]. Tan Lap cave was visited three times for this study, and samples were collected both in the wet (July 2017 and September 2018) and dry seasons (April 2017; Table 2). In Tan Lap cave, two annual birth periods of *Chaerephon plicatus* have been recorded, with parturition occurring in approximately April and October [56], as in other countries in the region [31]. The samples collected in April 2017 were therefore included in the birth period. No samples were collected in October, during the second annual birth period (Table 2).

### 2.7. Human Serology

Magnetic bead-based immunoassays based on viral recombinant proteins or virus-like particles (VLP) used as capture agents for anti-viral antibodies [57,58,59] were used to test 30 human serum samples for the presence of IgM and IgG antibodies against the following virus groups: Crimean–Congo hemorrhagic fever virus (CCHFV), hantaviruses (HNTV), Lassa fever virus (LASV), Rift Valley fever virus (RVFV), Ebolaviruses (EBOV), Marburg virus (MARV), Chikungunya virus (CHIKV) for the panalphavirus assay and Dengue virus 2 (DENV2) for the panflavivirus assay.

For IgG and IgM testing, all samples were run in a direct format at a 1:100 dilution in triplicate, using the appropriate detector conjugate (either anti-human IgG-PE or anti-human IgM-PE, all from commercial sources). The MAGPIX outputs results in median fluorescence intensity (MFI) for all agents in the panel for each sample run and sample triplicates were averaged for interpretation. All plates incorporated both negative and positive controls. The negative control serum was a known negative American serum, and the positive control serum was a mixture of serum from known IgG or IgM positive samples for each etiologic agent. The IgM assay excluded EBOV and MARV from the panel, as positive control sources were not available for comparison for these pathogens.

Once the samples were processed, quality control checks were performed to ensure a sufficient number of magnetic beads were read for each sample and to ensure intra-sample variation (within the sample triplicates) were within acceptable limits. Samples failing quality control checks were retested. Data were analyzed by calculating a signal to noise value by dividing the sample MFI value by the negative control MFI value for each agent. A sample was considered IgG or IgM positive if above a designated signal to noise cutoff based on observed variation for the negative and positive controls. A signal/noise cutoff of greater than or equal to 20× the negative control value was identified for positive samples for IgG testing to minimize false positives. A signal/noise cutoff of greater than or equal to 10× the negative control value was identified for positive samples for the IgM testing. Samples were considered negative if the signal/noise value fell below 4× the negative control value for both IgG and IgM. Samples with signal/noise values between the positive and negative cutoffs were considered suspect positives.

## 3. Results

### 3.1. Virus Detection in Bat and Pig Samples

A total of 1676 samples from bats, pigs and humans were screened for CoVs, while 1436 samples were screened for PmVs, influenza A virus, FlaviVs and FiloVs (Table 1 and Table S1). CoVs were detected in a large proportion of bat (30.5%) and pig (24.7%) samples, mostly in feces (32.7%) and rectal swabs (18.2%) for bats and in oral swabs (40%) for pigs (Table 1). A smaller proportion of samples, similar for bats (1.9%) and pigs (2.1%), were positive for PmVs. The proportion of bat samples positive for PmVs was larger for urine samples (16%) than for other sample types. No bat samples were positive for influenza A virus, but influenza A virus was detected in 4.9% of pig samples. No human samples were positive for any of the tested viral families.

Twenty-one bat samples were co-infected by two coronaviruses. These co-infections included PREDICT_CoV-17 with bat coronavirus 512/2005 (n = 1) and PREDICT_CoV-35 with bat coronavirus 512/2005 (n = 20) (Table S1). Five pig samples collected in 2018 were co-infected by Alphacoronavirus 1 and Betacoronavirus 1 (Table S2). Interestingly, most PmVs identified in bat feces and guano (12/13; PREDICT_PmV-13 (n = 4), PREDICT_PmV-63 (n = 3), PREDICT_PmV-66 (n = 5)) were detected in co-infection with bat coronavirus 512/2005.

Of the 539 bat samples additionally screened specifically for Sarbecoviruses and SARS-CoV-2, only one fecal sample (0.2%) from Tan Lap cave was positive for the RT-PCR targeting the E gene but was negative for the RT-PCR targeting the RdRp gene. A positive PCR product was then obtained from that sample when using the primers designed to target viruses related to pangolin Sarbecoviruses [43]. However, after sequencing, this virus was identified as an alpha-CoV (porcine epidemic diarrhea virus, PEDV), therefore indicating that these primers were not specific to Sarbecoviruses but were also able to amplify more distantly related CoVs. 

### 3.2. Phylogenetic Trees

**Figure 2:**
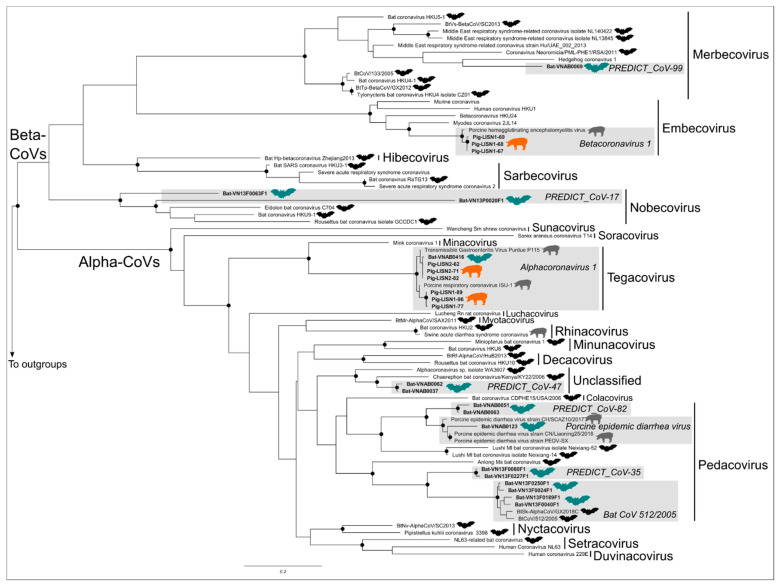
Maximum Likelihood phylogenetic tree summarizing phylogenetic relationships among bat and pig coronavirus RdRp sequences (concatenated dataset including two fragments of the RdRp gene) identified in Viet Nam. Well-supported nodes (bootstrap > 75%) are indicated by a black dot. Virus sequences isolated in bat samples from Viet Nam are indicated by a bat symbol colored in green while bat viruses from other countries are black. Sequences isolated in pig samples in Viet Nam are indicated by a pig symbol colored in orange while pig viruses from other countries are grey.

Alpha-CoVs and Beta-CoVs belonged to two distinct clades in our CoV phylogenetic tree and formed well-supported monophyletic lineages in the tree (Figure 2). The tree was inferred from a 717 bp concatenated fragment of the RdRp gene, and all CoV subgenera currently recognized by the ICTV, except the *Setracovirus* and *Pedacovirus* subgenera, were monophyletic. Two known and widespread CoVs, the Betacoronavirus 1 and the Alphacoronavirus 1, were detected in our pig samples collected in Viet Nam (Figure 2). Eight distinct CoV taxonomic units, three of them corresponding to known viruses and the other five to viruses first discovered by the PREDICT project, were identified in our bat samples (Table 1). Two of these CoV taxonomic units, named PREDICT_CoV-99 and PREDICT_CoV-17, belong to the *Merbecovirus* and *Nobecovirus* subgenera, respectively, within the Betacoronavirus genus, with PREDICT_CoV-99 being distantly related to MERS-CoV within the *Merbecorvirus*. The remaining six CoV taxonomic units, named Alphacoronavirus 1, PREDICT_CoV-47, PREDICT_CoV-82, PEDV, PREDICT_CoV-35 and bat CoV 512/2005, belonged to the *Tegacovirus* and *Pedacovirus* subgenera and an unclassified lineage within the Alpha-CoV genus (Figure 2). PREDICT_CoV-47, PREDICT_CoV-82, PREDICT_CoV-99 and PEDV were identified in Tan Lap cave in northern Viet Nam, where all samples we barcoded belonged to *Chaerephon plicatus*. PREDICT_CoV-17, PREDICT_CoV-35, Alphacoronavirus 1 and bat CoV 512/2005 were detected in the samples collected in the guano farms from Soc Trang and Dong Thap provinces in southern Viet Nam, where all barcoded samples were identified as *Scotophilus kuhlii.* There were no bat CoVs identified in both regions.

Only one virus, the Alphacoronavirus 1, was detected in both bat and pig samples from Viet Nam. PEDV, another swine virus, was also identified in our bat samples but was not detected in our pig samples from Viet Nam. Several of the other viruses identified in bats, PREDICT_CoV-82, PREDICT_CoV-35, and bat CoV 512/2005, are all closely related to PEDV and belong to the same subgenus (*Pedacovirus*) (Figure 2).

**Figure 3:**
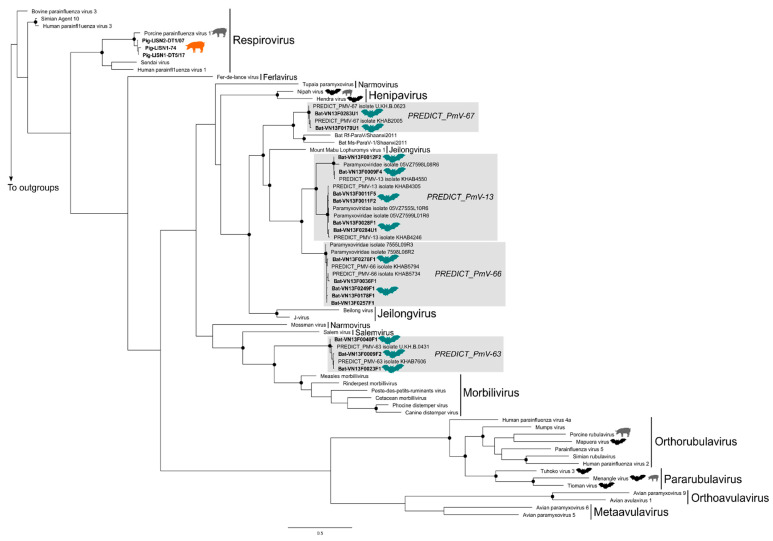
Maximum Likelihood phylogenetic tree summarizing phylogenetic relationships among bat and pig paramyxovirus Pol gene sequences identified in Viet Nam. Well-supported nodes (bootstrap > 75%) are indicated by a black dot. Virus sequences isolated in bat samples from Viet Nam are indicated by a bat symbol colored in green, while bat viruses from other countries are black bat. Sequences isolated in pig samples in Viet Nam are indicated by a pig symbol colored in orange while pig viruses from other countries are grey.

Internal nodes of our PmV phylogenetic tree were poorly supported but all PmV subgenera currently recognized by the ICTV, except the *Orthorubulavirus* subgenus, formed well-supported monophyletic lineages in the tree (Figure 3). Only one PmV, the porcine parainfluenza virus 1 belonging to the *Respirovirus* subgenus, was detected in our pig samples from Viet Nam while four distinct PmV taxonomic units, all corresponding to novel viruses, were identified in our bat samples (Table 1 and Figure 3). These four PmVs were only detected in samples collected in southern Viet Nam (Soc Trang and Dong Thap provinces). PREDICT_PmV-63 is closely related to the Morbilivirus subgenus, which includes Measles, Rinderpest and Peste-des-petits-ruminants viruses (Figure 3). The other bat PmVs identified in this study, PREDICT_PmV-13, PREDICT_PmV-66, and PREDICT_PmV-67, are closely related to viruses belonging to the *Jeilongvirus* subgenus (Figure 3). None of the bat PmVs identified in this study are phylogenetically related to PmVs known to cause significant diseases in pigs.

### 3.3. Phylogeography of Pig Viruses in Viet Nam

**Figure 4:**
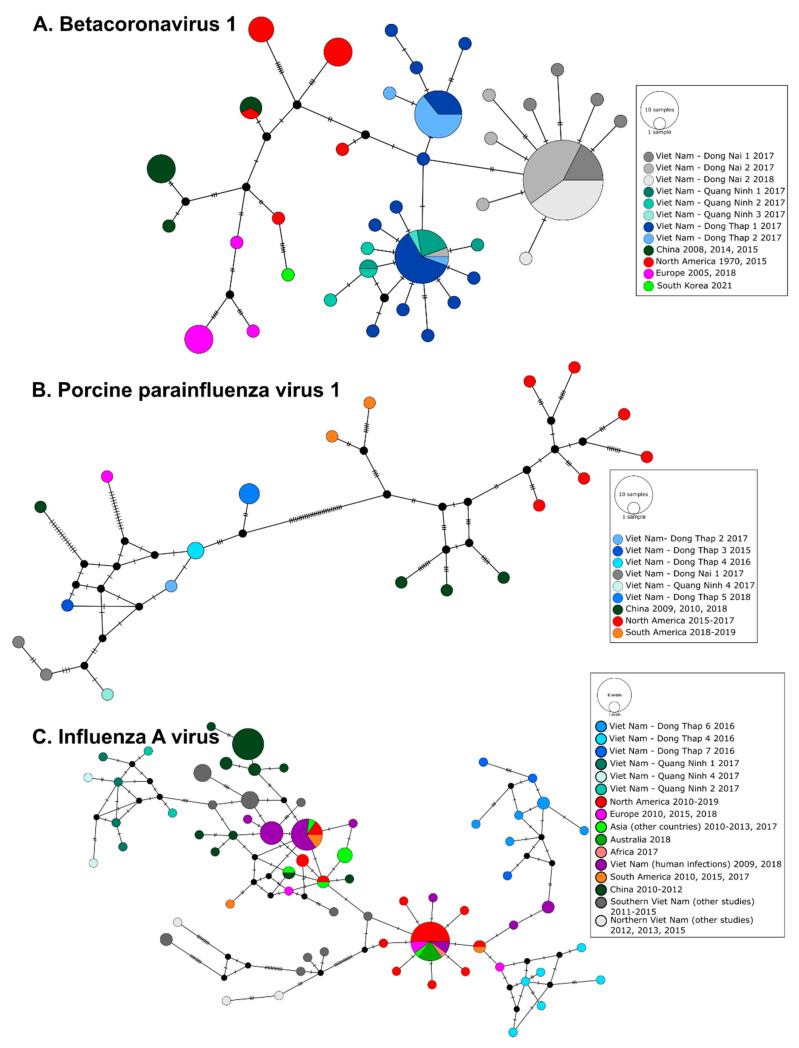
Median-joining networks of (**A**) Betacoronavirus 1 (RdRp gene, [36], 393 bp), (**B**) porcine parainfluenza virus 1 (Pol gene, 546 bp), and (**C**) influenza A virus (PB1 gene, 384 bp). Circles correspond to distinct viral sequences and circle sizes are proportional to the number of identical sequences in the dataset. Small black circles represent median vectors (ancestral or unsampled intermediate sequences). The numbers of mutational steps between sequences are represented as hatch marks along branches.

For all three pig viruses whose phylogeographic structure were investigated using median-joining networks, viral sequences detected in Viet Nam by this study were clearly genetically distinct from sequences obtained from domestic pigs in other regions of the world or in Viet Nam during similar time periods or a few years earlier (Figure 4). BetaCoV1 RdRp sequences showed a weaker phylogeographic structure than the other two swine viruses included in the analysis, porcine parainfluenza virus 1 and influenza A virus. However, all but one BetaCoV1 RdRp sequence from Dong Nai province obtained in 2017–2018 are clearly demarcated from sequences from Dong Thap province, despite their close geographical proximity (approximately 140 km) (Figure 4A), while sequences from farms located in Dong Thap and Quang Ninh provinces were very closely related, despite the geographic distance between these two provinces (i.e., approximately 1200 km). Viet Nam PPV1 sequences (Pol gene) also showed a marked geographic structure. They were distinct from sequences isolated in the Americas and China and were only distantly related to sequences from China and Germany (Figure 4B).

The swine IAV PB1 sequences from Quang Ninh province in northern Viet Nam were highly divergent from those found in Dong Thap province during this study and did not share similar ancestry (Figure 4C). Sequences isolated in Quang Ninh were related to older PB1 fragments from swine IAV in southern Viet Nam (Ho Chi Minh City, Tien Giang and Dong Nai provinces) derived from the pandemic A(H1N1)2009 virus (pdm09) [60] while those identified in Dong Thap were related to European swine AIV PB1 sequence (Dong Thap 4) and Viet Nam human AIV PB1 sequences (Dong Thap 6 and 7). IAV strains infecting humans in Viet Nam in 2009 and 2018 were not related to the strains we identified in pigs in 2016 and 2017 (Figure 4C).

### 3.4. Phylogeography and Spatio-Temporal Spread of Bat CoVs

**Figure 5:**
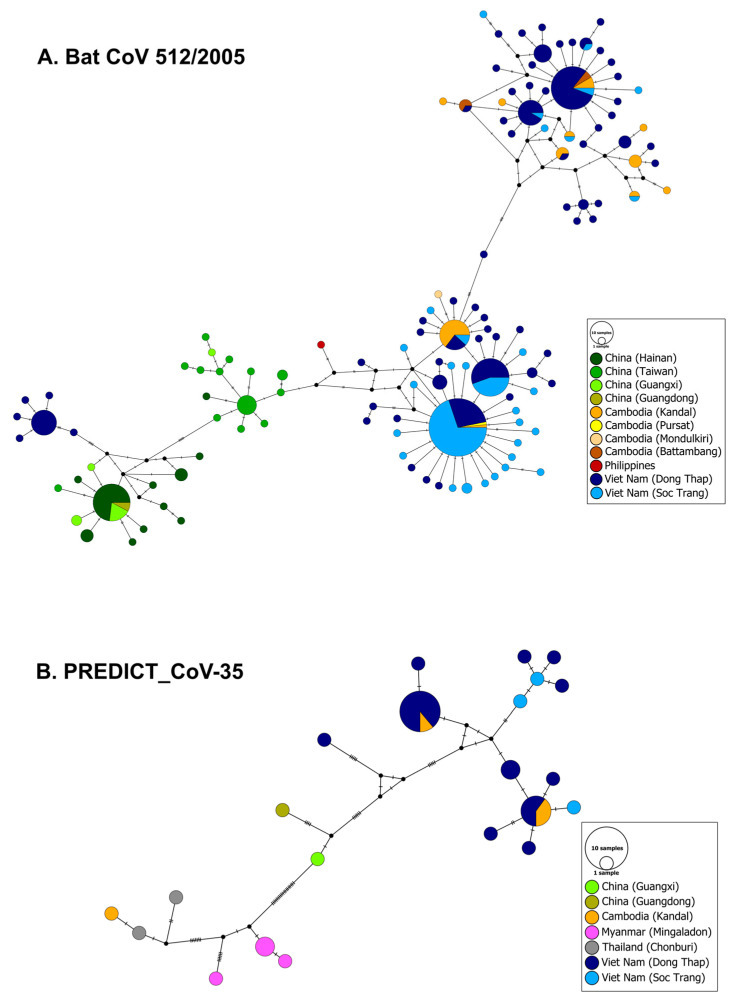
Median-joining networks of (**A**) bat CoV 512/2005 (RdRp gene, [36], 393 bp), and (**B**) PREDICT_CoV-35 (RdRp gene, [36], 393 bp). Circles correspond to distinct viral sequences and circle sizes are proportional to the number of identical sequences in the dataset. Small black circles represent median vectors (ancestral or unsampled intermediate sequences). The numbers of mutational steps between sequences are represented as hatch marks along branches.

The median-joining networks showed weaker phylogeographic structure for bat CoV 512/2005 and PREDICT_CoV-35 than for pig viruses (Figure 5). For both of these bat viruses, sequences from southern Viet Nam and Cambodia are highly similar and very closely related, and no geographic structure is detectable in the region. Only a few bat CoV 512/2005 sequences from Dong Thap are divergent from the other sequences isolated in the province and are linked to sequences from China (Figure 5).

**Figure 6:**
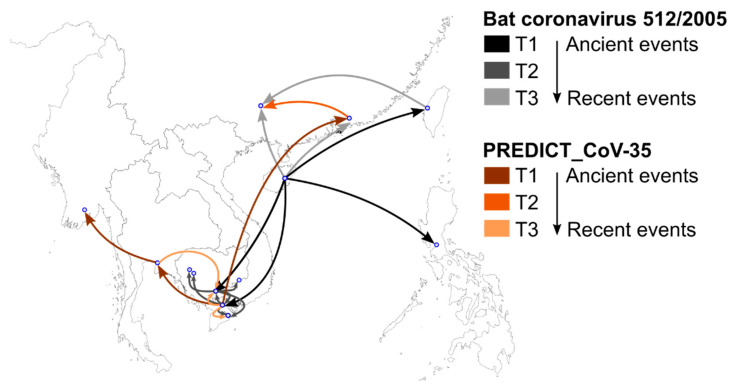
Spatiotemporal dispersal of bat coronavirus 512/2005 (grey shades) and PREDICT_CoV-35 (brown shades) in Southeast Asia inferred from a fragment of the RdRp gene (386 bp, [36]). Arrows indicate the direction of dispersal routes. Darker arrow colors indicate older dispersal events for both viruses.

The spatiotemporal dynamics of bat CoV 512/2005 and PREDICT_CoV-35 inferred from our BEAST discrete phylogeographic model showed a northeast/southwest demarcation in the origin and dispersal of these two viruses (Figure 6). Bat CoV 512/2005 likely originated in Hainan, China, from where it dispersed southwards and eastwards. Numerous dispersal events were then inferred among locations in southern Viet Nam and Cambodia. The most recent inferred dispersal movements of bat CoV 512/2005 occurred from Hainan and Taiwan to mainland China. The inferred origin of PREDICT_CoV-35 was located in southern Viet Nam, from where the virus dispersed to the west, to Thailand and Myanmar, and to the north in China (Figure 6). More recent dispersal movements of PREDICT-CoV-35 occurred in the southern part of the Indochina region.

### 3.5. Seasonal Effect on CoV Detection in Bat Guano

The proportion of positive guano samples in the bat guano farms in southern Viet Nam was higher during the wet season (45.8% (291/635), 95% CI 43–49%) than the dry season (28.3% (32/113), 95% CI 26–31%) and during the “no birth period” (63.4% (265/418), 95% CI 60–66%) than the “birth + lactation period” (17.6% (58/330), 95% CI 15–20%). The opposite trend was observed in the bat cave in northern Viet Nam where a higher proportion of positive samples was observed in the dry season (12.2% (9/74), 95% CI 10–14%), which also corresponds to the birth period, than in the wet season (3.1% (7/226), 95% CI 2–4%). 

Our univariate models confirmed that season and reproductive period were highly significantly associated with the detection of bat CoV but with opposite effects in both sites. The wet and “no birth” seasons were associated with significantly higher probability of CoV detection in samples from the guano farms of southern Viet Nam (OR = 2.14, 95% CI 1.39–3.36 and OR = 8.12, 95% CI 5.78–11.56, respectively) and significantly lower probability of detection in samples from the guano cave in northern Viet Nam (OR = 0.23, 95% CI 0.08–0.64).

### 3.6. Human Serology

Of the 30 human serum samples tested, 26 samples (86.7%) had detectable IgG antibodies against flaviviruses (Table 3 and Table S6). IgG antibodies against CCHFV were detected in one sample and against MARV in another sample (Table 3 and Table S6). None of the samples had detectable IgM antibodies against any of the targeted viruses (Table 3 and Table S6).

## 4. Discussion

The One Health cross-sectoral surveillance approach utilized in this study identified the circulation of viruses with zoonotic potential in bats, pigs, and humans in Viet Nam. It provides a concrete example of implementing One Health surveillance in collaboration with national animal health and public health institutions in a region known as a “hotspot” for emerging infectious diseases [61]. A multi-stakeholder workshop approach proved effective for identifying sampling sites and designing the cross-sectoral study at the national level. Additional coordination was required at the provincial and district level to implement concurrent surveillance in wildlife, domestic animal, and human populations. The designated surveillance periods occasionally required adjustment to accommodate the need for the public health and animal health institutions to respond to on-going disease outbreaks such as Foot and Mouth Disease in livestock, and Dengue fever outbreaks in humans. The animal health institutions and associated animal health laboratories in Viet Nam have a mandate for investigating disease and screening for infectious agents in all animals, which facilitated the inclusion of wildlife species in their surveillance activities and adaptation of sampling and testing protocols to include wildlife and consensus PCR approaches. The public health institutions in Viet Nam have a mandate for investigating wildlife reservoirs of known zoonotic pathogens such as plague, but investigating viruses of pandemic potential in wildlife species falls in the domain of research. This made it challenging to incorporate screening for novel viruses into established national influenza and hospital-based severe acute respiratory illness (SARI) surveillance programs. Although technically feasible to expand the screening of specimens collected through hospital-based SARI surveillance, there was resistance to changing sampling and testing protocols due to recent national harmonization efforts which in turn limited the application of the intended consensus PCR testing approach. Despite the challenges encountered in coordinating concurrent cross-sectoral surveillance, and the limited results from some of the targeted populations, our study revealed a diversity of viruses with zoonotic potential and demonstrated the feasibility of conducting surveillance across wildlife–livestock–human spillover interfaces generating important data for integrated analyses for epidemiological risk assessment, which has been identified as a critical need for pandemic prevention and preparedness [7].

Significant diversity, with eight CoVs and four PmVs, was detected in bats roosting at the human–animal interfaces targeted for this study. These bat communities, including *Scotophilus kuhlii* in guano farms of southern Viet Nam and *Chaerephon plicatus* in the cave of northern Viet Nam, were characterized by distinct viral diversity, as none of the viruses identified in this study were detected in both communities. These two bat species belong to two distinct families, Vesperstilionidae and Molossidae, respectively, within the Yangochiroptera, which may explain this divergent viral diversity and is consistent with the strong association between CoV diversity and host taxonomy observed in the region [11,62]. A majority of CoVs and PmVs detected in this study were novel viruses first discovered by the PREDICT project but are not restricted to Viet Nam. PREDICT_CoV-47 and PREDICT_CoV-82 were also detected in Thailand and Myanmar, respectively [33,63]. PREDICT_CoV-17, PREDICT_CoV-35 and bat CoV 512/2005 were previously identified in several other countries of Southeast Asia [33,62,63]. The four PmVs detected in this study were also previously found in Cambodia and/or Viet Nam [33,64].

Bats are known to host some high consequence zoonotic pathogens and their progenitors within the CoV and PmV families, such as SARS-CoV-1, SARS-CoV-2, MERS-CoV, Nipah virus and Hendra virus [20]. However, the zoonotic spillover potential of the bat viruses identified in this study remain mostly unknown, as these viruses have not been fully characterized and their ability to jump species barriers has not been assessed. PREDICT_CoV-99 is of particular interest, as it is related to MERS-CoV within the *Merbecorvirus* and it has not been found in another country previously. Whole genome sequencing of PREDICT_CoV-99 was attempted during this study, but no sequence longer than the one included in this study could be obtained from this sample.

Bat CoV 512/2005 was the most prevalent virus in our bat sample set (294/494 viral detections, 59.5%), followed by PREDICT_CoV-35 (45/494 viral detections, 9.1%). According to a risk ranking framework for wildlife viruses based on risk factors that are most likely to contribute to spillover risk [65], PREDICT_CoV-35 presents a high zoonotic risk score. This ranking is due to its broad host and virus distribution combined with numerous detections in bats at high-risk disease transmission interfaces, characterized by high land use modifications and frequent interactions among wildlife, domestic animals and humans. Bat CoV 512/2005 ranked lower due its more restricted distribution range but still has a high-risk score because of its association with anthropogenic and fragmented environments with high human and livestock density [65,66]. These findings suggest that PREDICT_CoV-35 and bat CoV 512/2005 are of high public health relevance in southern Viet Nam where we detected them. Our phylogeographic inference showed that these viruses are circulating widely in Asia and frequently dispersing across borders, illustrating the widespread potential risk of zoonotic spillover from bat viruses that is not limited to a single country in the region. Co-infection involving these two viruses were also frequently detected in our bat samples, which may facilitate virus recombination or reassortment and the emergence of viral strains with high zoonotic potential [67].

Two known and widespread CoVs, BetaCoV1 and AlphaCoV1, and one PmV, PPV1, were detected in pig farms sampled in Viet Nam. AlphaCoV1 was also detected in a bat sample from a guano farm in Dong Thap. PEDV, another swine virus originating from bats [68,69], was also identified in a bat sample collected in the cave in Lang Son province but was not detected in our pig samples, despite the confirmed circulation of the virus in pig populations in northern Viet Nam in 2018–2019 [70]. These findings illustrate the high risk of CoV spillover from bats to pigs in Viet Nam, where pig density is very high. The risk related to PmV seems to be lower, as none of the bat PmVs detected in this study are related to known pig pathogens.

The marked phylogeographic structure of swine PPV1 and IAV indicated localized viral transmission among pig farms in Viet Nam and did not show large scale spread of swine viruses in the country. Our networks for these two viruses showed that similar or closely related strains circulated in the same provinces over several years and did not seem to spread to other regions. However, the lack of HA and NA genes from our IAV positive samples is a big limitation of this study and prevents us from fully characterizing these IAV strains and reassortment patterns. These findings corroborate previous studies suggesting multiple introductions, some of them resulting from human-to-pig IAV spillovers and co-circulation of divergent strains of swine IAV in Viet Nam [29,60]. The small-scale supply chain and limited pig movements in the country may explain the lack of widespread gene flow and marked phylogeographic structure observed for these two viruses in this study [9,71]. However, not all pig viruses are characterized by a similar phylogeographic pattern, as BetaCoV1 showed some larger scale viral sharing with the same strain detected in both northern and southern Viet Nam (Figure 4). It should be emphasized that these results were obtained from short gene fragments, whole genome sequencing is required to confirm these findings and fully characterize the evolution and circulation of these viruses.

The end of the wet season (October), a few months after the birth and lactation period, is the period when we observed the highest CoV prevalence in bats in southern Viet Nam. This would corroborate findings from Cambodia showing higher CoV circulation in juveniles and young bats after they entered the general population [72]. Increased CoV shedding in juvenile bats becoming independent was also detected in East-African bats [73]. We observed an opposite pattern in northern Viet Nam, where the highest prevalence was detected at the end of the dry season which is also when births occur. However, our limited multi-year sampling does not allow us to disentangle the impact of both seasonality and inter-annual cycles on CoV prevalence and circulation in bat populations of northern and southern Viet Nam. Longitudinal surveillance is needed to confirm these observations [74].

These findings have important public health implications for populations involved in guano farming and harvesting in Viet Nam. When considering health a global good, they indicate that guano collection, if not stopped entirely, should at the very least be avoided during periods of high CoV prevalence and the use of personal protective equipment must also be encouraged for guano harvesters. In addition to health costs, the impact of bat guano harvesting on the conservation of bats and potential disruption of their critical role in the broader ecosystem as pollinators and consumers of pathogen-carrying arthropods and agricultural pests must be assessed and costed out to “... sustainably balance and optimize the health of people, animals, and ecosystems” [5].

Our very limited human sampling failed to detect the spillover of bat zoonotic viruses into human communities living close to the bat cave and harvesting bat guano. Longitudinal surveillance, including during periods of high CoV prevalence in bats, and much larger sample sizes would be needed to detect these events. However, our serological study detected possible previous exposure to Marburgvirus-like (Filoviridae) and Crimean–Congo hemorrhagic fever virus-like (Bunyaviridae) viruses. Even if cross-reaction with other viruses cannot be ruled out, these findings suggest that some potentially high-impact viruses are circulating in these communities in contact with wildlife. The high flavivirus IgG seropositivity rate in this population is most likely linked to exposure to the mosquito-borne dengue virus and reflects the country’s high rates of dengue infections. Viet Nam has one of the highest dengue morbidity rates in the world with well over 300,000 cases reported in 2022 [75,76]. Similarly, the detection of alphaviruses IgG seropositivity could be associated with exposure of Chikungunya virus which is also prevalent in the region [77]. Although all our bat samples were negative for flaviviruses, it should also be noted that several flaviviruses have been detected in bats worldwide, including Japanese encephalitis virus [78,79], Usutu virus [80,81] and West Nile virus [82].

This study represents one of the first attempts to implement One Health surveillance in Viet Nam which is located within a region characterized as a global hotspot for emerging infectious diseases. Surveillance was targeted at locations, with respective practices and behaviors, where spillovers are most likely to occur and coordinated to include data and sample collection from wildlife species, domestic animals, and humans. Despite its limitations, our study highlights circulation of viruses with zoonotic potential in bats, pigs and humans in Viet Nam, the high risk of CoV spillover from bats to pigs, and opportunities to improve the efficiency of One Health surveillance in emerging infectious disease hot spot countries. Coordinating surveillance in wildlife with on-going national pathogen-specific surveillance programs in livestock proved effective and efficient as a mechanism to expand the number of domestic animal specimens screened for viruses of pandemic potential and facilitate analysis of data across wildlife and domestic animal populations. Integrating hospital-based SARI surveillance in humans into a One Health surveillance approach in Viet Nam showed promise; however, stakeholder resistance to adapting sampling and testing protocols given recent investments in harmonizing the national SARI surveillance program, coupled with bureaucratic challenges, limited the full application of the intended consensus PCR testing and sequencing approach which in turn greatly reduced the data available from humans for our analyses. The growing recognition of the pandemic potential of emerging coronaviruses post-COVID-19 has already led many public health agencies to expand their SARI and related surveillance programs beyond influenza. There remains a need to integrate wildlife expertise into One Health surveillance planning and expand knowledge of wildlife systems (free range populations of wildlife, wildlife farming operations, and wildlife trade) to inform One Health surveillance approaches broadly. Wildlife knowledge will be critical in the targeting of surveillance locations, interpretation of surveillance data, and development of interventions needed to prevent novel virus emergence as well as prepare for and respond effectively to the emergence of viruses with pandemic potential.

## Figures and Tables

**Table 2:** Summary of positive samples by site, sampling month and sampling season.

Guano Farms in Southern Viet Nam				
Month (Year)	Season	Samples Tested	Samples Positive	Percentage Positive
January (2013)	Dry; no birth	8	1	12.5%
June (2018)	Wet; birth + lactation	130	15	11.5%
July (2017)	Wet; birth + lactation	200	43	21.5%
October (2013)	Wet; no birth	305	233	76.4%
December (2017)	Dry; no birth	105	31	29.5%
Bat cave in northern Viet Nam				
Month (Year)	Season	Samples Tested	Samples Positive	Percentage Positive
April (2017)	Dry; birth	74	9	12.2%
July (2017)	Wet; no birth	126	7	5.5%
September (2018)	Wet; no birth	100	0	0%

**Table 3:** Seroprevalence of IgM and IgG antibodies of each target virus among bat guano harvesters and community members in Huu Lung district. Number of samples testing positive/number of suspect positives/total number tested (and percent tested positive) by MAGPIX.

Target Virus	IgG	IgM
	Positive/Suspect/Total (% Positive)	Positive/Suspect/Total (% Positive)
Alphaviruses (CHIKV)	0/1/30	0/1/30
Crimean–Congo hemorrhagic fever virus (CCHFV)	1/5/30 (3.3 %)	0/0/30
Flaviviruses (DENV2)	26/3/30 (86.7 %)	0/0/30
Hantaviruses (HNTV)	0/5/30	0/0/30
Lassa fever virus (LASV)	0/2/30	0/2/30
Rift Valley fever virus (RVFV)	0/4/30	0/1/30
Ebolaviruses (EBOV)	0/3/30	NA
Marburg virus (MARV)	1/0/30 (3.3 %)	NA

## Data Availability

All relevant data are available as supplementary materials.

## Supplementary Materials

The following supporting information can be downloaded at: https://www.mdpi.com/article/10.3390/v15030790/s1, Table S1: PREDICT dataset including all bat, pig and human samples included in this study and their testing results for five viral families including coronaviruses, paramyxoviruses, influenza viruses, filoviruses and flaviviruses and GenBank accession numbers for sequences detected in this study. Table S2: LISN dataset including positive testing results of 120 pig samples from Dong Nai and Dong Thap provinces collected in 2018 and tested for five viral families including coronaviruses, paramyxoviruses, influenza viruses, filoviruses and flaviviruses and GenBank accession numbers for sequences detected in this study. Table S3: GenBank accession numbers for coronavirus reference sequences used in our phylogenetic analyses, spatiotemporal dispersal analysis and median-joining networks. Table S4: GenBank accession numbers for paramyxovirus reference sequences used in our phylogenetic analyses and median-joining networks. Table S5: GenBank and GISAID accession numbers for influenza A reference sequences used in our phylogenetic analyses and median-joining networks. Table S6: Results of magnetic bead-based immunoassays used to test 30 human serum samples for the presence of IgM and IgG antibodies against the following virus groups: Crimean–Congo hemorrhagic fever virus (CCHFV), hantaviruses (HNTV), Lassa fever virus (LASV), Rift Valley fever virus (RVFV), Ebolaviruses (EBOV), Marburg virus (MARV), alphaviruses and flaviviruses. Table S7: GISAID acknowledgement table.

## Appendix A

The list of individuals in the PREDICT Consortium is available at https://ohi.sf.ucdavis.edu/programs-projects/predictproject/authorship (accessed 17 March 2023) and includes Ola Ababneh, Mustafa Ababneh, Jum Rafiah Abd Sukor, Mohd Lutfi Abdullah, Josefina Abedin, Ehab Abu-Basha, Mohamed Ahmed Ali, Junior Beal Akoundze, Joël Akpaki, Sief Addeen Al Hanandeh, Bilal Al Omari, Abdullah Al Shakil, Mohammad Borhan Al-Zghoul, Stephenie Ann Albart, Abdullah Alshammari, Basil H. Amarneh, William Ampofo, Victoria Andrew, Dao Le Anh, Ulaankhuu Ankhanbaatar, Simon Anthony, Ungke Antonjaya, Kidan Araya, Jallah Arku, Norsharina Arshat, Theodore Asigbee, Ohnmar Aung, Joseph Awuni, James Ayukebong, Mohamed Saifullah Mohd Azian, Nor Adilah Aziz, Aminata Ba, Ganzorig Baasan, Zena Babu, Ola Bagato, Aboubacar Bamba, Djeneba Bamba, James Bangura, Ariunbaatar Barkhasbaatar, June Barrera, Cale Basaraba, Samuel Bel-Nono, Manjunatha Belaganahalli, Desalegn Belay, Jaber Belkhiria, Ridzki M.F. Binol, Brian Bird, Manisha Bista, Pitu Biswas, Matthew Blake, Linda Boatemaa, Margret Bonason, James Brandful, Joseph Brown, John Brownstein, Alpha Oumar Camara, Mamadi Camara, Salif Camara, Daniel Chai, Debapriyo Chakraborty, Hannah Chale, Ashok Chaudhary, Sokha Chea, Aleksei A. Chmura, Andrew Chow, Carolina Churchill, Abraham Commey, Emmanuel Couacy-Hymann, Julien Kalpy Coulibaly, Michael Cranfield, Wirda Damanik, Batchuluun Damdinjav, Norhidayah Danial, Peter Daszak, Runie David, Patrick Dawson, Ardjouma Dembele, Awa Deme, James Desmond, Aghnianditya Kresno Dewantari, Jasjeet Dhanota, Tapan Dhole, Nguyen Thi Diep, Aristide Dionkounda, Gaye Laye Diop, Kimberly Dodd, Otilia Dogbey, Tumendemberel Dorjnyam, Mireille Dosso, Kalil Doumbouya, Mohamed Idriss Doumbouya, Megan Doyle, Simone Dramou, Tracy Drazenovich, Dang Duc Anh, Bach Duc Luu, Prateep Duengkae, Vu Trong Duoc, Tran Nhu Duong, Veasna Duong, Huda Dursman, Philippe Dussart, Shusmita Dutta, Tan Jun Ee, Abel Ekiri, Amira S. El Rifay, Rabeh El Shesheny, Ahmed N. El Taweel, Zena Emmanuel, Jonathan H. Epstein, Jason Euren, Tierra Smiley Evans, Alaa Fahmawi, Simeon Fahn, Yasha Feferholtz, Jinnat Ferdous, Amanda Fine, Meerjady Sabrina Flora, Leilani Francisco, Lem Fui Fui, Taylor Gabourie, Millawati Gani, Michael Garbo, Nicole Gardner, Aiah Gbakima, Marie Pelagie Atrou Gbamele, Xingyi Ge, Lee Heng Gee, Brooke Genovese, Alexandra Gibson, Kirsten Gilardi, Martin Gilbert, Amethyst Gillis, Andrew Ginsos, Privat Godji Gnabro, Tracey Goldstein, Mokhtar R. Gomaa, Jules Gomis, Kevin Gonzalez, Zoe Grange, Denise Greig, Michael Grodus, Kpon Kakeuma Romeo Gueu, Leticia Gutierrez, Dan Marcelin Haba, Emily Hagan, Abdul Hai, Suraya Hamid, Daniel K. Harris, Abdul Kadir Abu Hashim, Moushumi Hassan, Quaza Nizamuddin Hassan, Qun He, Thiravat Hemachudha, Helena Henry, Ronald Herbert, Zaidoun Hijazeen, Moukala Ndolo Hilarion, Rebecca Hill, Nguyen Thi Hoa, Paul Horwood, Md. Enayet Hossain, Saddam Hossain, Moh Moh Htun, Ben Hu, Tom Hughes, Vibol Hul, Vo Van Hung, Fatima Hussein, Ghislain Dzeret Indolo, Diah Iskandriati, Ariful Islam, Md. Tarikul Islam, Shariful Islam, Mohd Isnaim Ismail, Zuhair Bani Ismail, Jacques Iyanya, Joel Judson Jaimin, Amara Jambai, Jeffrine Rovie Ryan Japning, Alexter Japrin, Frantz Jean Louis, Titus Joe, Christine K. Johnson, Erica Johnson, Damien Joly, Jyotsna Joshi, Jusuf Kalengkongan, Douokoro Kalivogui, Nenneh Kamara-Chieyoe, Joseph Kamau, Eddy Kambale Syaluha, Ahmed Kandeil, Yagouba Kane, William Karesh, Kandeh Kargbo, Dibesh Karmacharya, Novie Kasenda, Ghazi Kayali, Ahmed S. Kayed, Rudovick Kazwala, Changwen Ke, Lucy Keatts, Nigatu Kebede, Nigatu Kebede, Bouaphanh Khamphaphongphane, Chong Chee Kheong, Christopher Kilonzo, Ma-Sue Koffa, Amos G. Kollie, Marcel Sidiki Kondiano, Michel Koropo, Valere Kouamé Kouakou, Eugene Kouassi Koffi, Mariam Kourouma, Abdoulaye Ousmane Koutate, Citra Livi Kowel, Hermann Assemien Krou, Charles Kumakamba, Tina Kusumaningrum, Omnia Kutkat, François Lamah, Nguyen Thi Lan, Jennifer Lane, Christian Lange, Emmanuel Larmouth, Alice Latinne, Anne Laudisoit, Joseph Diffo Le Doux, Elizabeth Leasure, Katherine Leasure, Mat LeBreton, Jimmy Lee, Helen Lee, Mei Ho Lee, Amara Leno, Hongying Li, Eliza Liang, Neal Liang, Dorothy Lim, W. Ian Lipkin, Jun Liu, Modou Moustafa Lo, Leonoris Lojivis, Nguyen Van Long, Nguyen Van Long, Ashley Lucas, Jean Paul Lukusa, Victor Lungay, Shongo Lushima, Julius Lutwama, Wenjun Ma, Catherine Machalaba, Grace Mwangoka, Walter Simon Magesa, Sara H. Mahmoud, Maria Makuwa, Asha Makweta, Abdullah Al Mamun, Prajwol Manandhar, Patarapol Maneeorn, Harjeet Mann, Pe Bhele Maomy, Victorine Maptue, Stephanie Martinez, Alice Mathew, Yanne Vanessa Mavoungou, Min Thein Maw, Jonna Mazet, Placide Mbala, Emmanuel Mbuba, Eric Mbunwe, David McIver, Emma Mendelsohn, Valchy Bel-Bebi Miegakanda, Maureen Miller, Phan Quang Minh, Happy Mkali, Yassmin Moatasim, Jean Vivien Mombouli, Corina Monagin, Diego Montecino-Latorre, Arsene Mossoun Mossoun, Ahmed Mostafa, Moctar Mouiche, Romain Bagamboula Mpassi, Alphonce Msigwa, Antoine Mudakikwa, Laura Benedict Mugok, Prime Mulembakani, Suzan Murray, Fakhrul Hatta Musa, Pacifique Musabimana, Samson Mutura, Tunu Mwamlima, Mwokozi Mwanzalila, Tin Tin Myaing, Theingi Win Myat, Aung Myo Chit, Magassouba N’faly, Manzan Jean N’Guettia, Anatole N’télo, Sylivia Nakimera, Vu Sinh Nam, Rajindra Napit, Senthilvel K.S.S. Nathan, Isamara Navarrete-Macias, Kortu M. Ndebe, Amadou Ndiaye, Daouda Ndiaye, Yohannes Negash, Nguyen Thi Thanh Nga, Ipos Ngay, Pham Thi Bich Ngoc, Fabien Niama, Rock Aimé Nina, Schadrack Niyonzima, Felix Nkom, Cynthia Nkoua, Noorliza Noordin, Rachmitasari Noviana, Julius Nwobegahay, Julius Nziza, Daniel O’Rourke, Tammie O’Rourke, Evangeline Obodai, Ricky Okello Okwir, Kevin Olival, Sarah Olson, Onkirotin Dionne Olva, Victoria Ontiveros, Fernandes Opook, Joko Pamungkas, Chandrawathani Panchadcharam, Pranav Pandit, Henri-Joseph Parra, Tran Minh Phuc, Nguyen Thanh Phuong, Jackson Y. Poultolnor, Saman Pradhan, Eunah Cho Preston, Mathieu Pruvot, Dulam Purevtseren, Dhiraj Puri, Le Tin Vinh Quang, Novie Rachmitasari, Kaisar Rahman, Mizanur Rahman, Mohammed Ziaur Rahman, Mustafizur Rahman, Diana Ramirez, Nistara Randhawa, Samita Raut, Joseph Rosario, Albert Ross, Noam Ross, Melinda Rostal, Pamela Roualdes, Eddy Rubin, Aftab Uddin Rumi, Christina Rundi, Melkor Sackie, Dodi Safari, Zikankuba Sijali, Sandra G. Samuels, Mathias Sango, Ammar Rafidah Saptu, Suryo Saputro, Daniel N’guessan Saraka, Alvis A. Sartee, Sia Alida Sayandouno, Karen Saylors, Mame Cheikh Seck, Victoria Sedor, Shahanaj Shano, Ajay Narayan Sharma, Velsri Sharminie, Mahmoud M. Shehata, Gafur Sheikh, Zhengli Shi, Enkhtuvshin Shiilegdamba, Bishwo Shrestha, Rima Shrestha, Mohammed Sidibey, Soubanh Silithammavong, Daniel Simon, Emilly Sion, Symphorosa Sipangkui, Frankie Thomas Sitam, Brett Smith, Bridgette Smith, Woutrina Smith, Batsikhan Sodnom, Benard Ssebide, Maria Suleiman, Ava Sullivan, Nur Amirah Md Sungif, Richard Suu-Ire, Mouhamed Sy, Jean Michel Takuo, Hani Talafha, Ubald Tamoufe, Emmanuel Tetteh, Aung Than Toe, Lanash Thanda, Ngo Thanh Long, Wai Zin Thein, Watthana Theppangna, Nguyen Duc Thinh, Hoang Bich Thuy, Nguyen Thu Thuy, Eri Togami, Moise Bendoua Tolno, Kevin Tolovou, Rahmat Topani, Alexandre Tremeau-Bravard, Ian Trupin, Jean Claude Tumushime, Kyaw Yan Naing Tun, Joseph Turay, Helal Uddin, Marcela Uhart, Nicole Ureda, Marc Valitutto, Khebir Verasahib, Megan Vodzak, Supaporn Wacharapluesadee, Mohammad Yuery Wazlan Abdul Wahad, Brooke Watson, Heather Wells, Allison White, Anna Willoughby, Ageng Wiyatno, David Wolking, Xinglou Yang, Lim Ming Yao, Sayon Yombouno, Cristin Young, Carlos Zambrana-Torrelio, Zahidah Izzati Zeid, Ghadeer Zghoul, Libiao Zhang, Yunzhi Zhang, Guangjian Zhu, Dawn Zimmerman, and Daba Zoumarou.
